# Hyperglycemic Challenge and Distribution of Adipose Tissue in Obese Baboons

**Published:** 2014-02-17

**Authors:** Tanmay Kulkarni, Gymama Slaughter, Chimdi Ego-Osuala, Peter Kochunov, Raul A. Bastarrachea, Vicki Mattern, Marcia Andrade, Paul B. Higgins, Anthony G. Comuzzie, V. Saroja Voruganti

**Affiliations:** 1 Bioelectronics Laboratory, University of Maryland, Baltimore County, Baltimore Maryland; 2 Maryland Psychiatric Research Center, University of Maryland School of Medicine, Baltimore, Maryland; 3 Research Imaging Institute, University of Texas Health Science Center at San Antonio, Texas; 4 Texas Biomedical Research Institute, San Antonio, TX; 5 Southwest National Primate Research Center, San Antonio, TX; 6 Center for Laboratory Animal Breeding, Oswaldo Cruz Foundation, Rio de Janeiro, RJ, Brazil; 7 Kunming Biomedical International, Kunming, PR China; 8 Department of Nutrition and Nutrition Research Institute, University of North Carolina at Chapel Hill, Kannapolis, NC

**Keywords:** Hyperglycemic Challenge, Perfusion Imaging, Body Fat Composition

## Abstract

**Background:**

Blood glucose levels regulate the rate of insulin secretion, which is the body’s mechanism for preventing excessive elevation in blood glucose. Impaired glucose metabolism and insulin resistance have been linked to excess body fat composition. Here, we quantify abdominal muscle and abdominal adipose tissue compartments in a large nonhuman primate, the baboon, and investigate their relationship with serum glucose response to a hyperglycemic challenge.

**Methods:**

Five female baboons were fasted for 16 hours prior to 90 minute body imaging experiment that consisted of a 20-min baseline, followed by a bolus infusion of glucose (500mg/kg). The blood glucose was sampled at regular intervals. The total volumes of the muscle, visceral and subcutaneous adipose tissue were measured.

**Results and discussion:**

We found that adipose tissue composition predicted fluctuations in glucose responses to a hyperglycemic challenge of a non-human primate. Animals with higher visceral adiposity showed significantly reduced glucose elimination. The glucose responses were positively correlated with body weight, visceral and muscle fat (p < 0.005). Polynomial regression analysis showed that body weight, visceral and muscle were significant

**Conclusions:**

These results reveal the similarity between humans and baboons with respect to glucose metabolism and strengthen the utility of baboon for biomedical research.

## Introduction

Obesity and its comorbidities such as type 2 diabetes and cardiovascular disease have become major health issues worldwide. Obesity is characterized by excess fat accumulation which contributes to the pathogenesis of these metabolic diseases. Central or abdominal adiposity is more closely associated with cardiometabolic risk than gluteofemoral obesity and is generally thought to worsen the metabolic disorders that are associated with overall obesity [1]. Fat in the abdominal area has a high turnover rate and is metabolically active. Individuals with abdominal or visceral fat accumulation had more obesity-related metabolic diseases than individuals with subcutaneous fat deposition [2]. In addition, dysregulation of this fat is known to cause a spillover of fat to other non-adipose organs such as liver, muscle, pancreas, etc resulting in increased metabolic disease risk and this can be independent of general adiposity [3]. Therefore, the distribution of fat seems to be more important than the amount of fat [4].

Given the effect of total adipose tissue distribution and composition, specifically abdominal fat, on the metabolic consequences of obesity [5], there is an urgent requirement for biologically relevant models to explain the role of abdominal obesity in metabolic disorders; nonhuman primate (NHP) models have potential for this purpose. The NHP and humans develop many similar metabolic disorders and NHP models for insulin resistance, hyperglycemia and atherosclerosis have greatly expanded our understanding of the genetic and environmental risk factors for these disorders [6]. Despite the inferred relevance of NHP research to human disease research, there has been a shortage of primate models available for biomedical research on metabolic disorders [6]. From a neuroimaging perspective, baboons offer the advantage of having among the largest brains of commonly studied laboratory primate [7]. Furthermore, our previous neuroimaging studies in baboons have demonstrated that the baboon model offers clinically relevant structural, functional, physiological and metabolic information about brain structure, function, development, and genetic variability [8–9]. The rationale of this study is to aid the development of NHP model to determine the effect of body fat composition on metabolic function by combining MRI technique with a commonly used metabolic challenge, the hyperglycemic challenge, to replicate previous findings in humans.

Techniques such as magnetic resonance imaging (MRI) and spectroscopy (MRS) are considered ‘gold standards’ for measuring fat content and distribution. Recent improvements in the MRI technology have enabled for non-invasive measurements of adipose tissue in the abdominal regions of the body. The measurement of abdominal adipose tissue (internal adipose tissue compartments) is extremely difficult to acquire with ultrasound or calliper techniques [10–11]. MRI techniques offer an advance mechanism to visualize and quantify adipose tissue in different regions of the body without exposing the subject to radiation as is the case with computerized tomography [10, 12]. MRI techniques can be easily used to distinguish adipose tissue from other tissue constituents because adipose tissue has a different proton relaxation time as compared to other tissues [12]. Here, we employed MRI as a method to quantify total adipose tissue (visceral, subcutaneous and muscle) and to accurately predict the effect of body fat composition on a hyperglycemic challenge (systemic glucose level): from fasting glycemia to a mild hyperglycemia.

## Materials and Methods

### Animal subjects and experimental protocol

A total of five adult (mean age = 9.17 ± 1.2 years [range: 8.4 – 11.7 years]) female baboons (Papio hamadryas Sp.) were selected from a large, breeding colony maintained by the Southwest National Primate e subjects had an fiv ResearchCenter(SNPRC). The average body weight of 26.7 ± 2.21 kg (range 23.25–28.56 kg). All animals had a stable weight pattern (< 3% change over the last 12 month) with normal euglycemic blood glucose values ([Glc] = 89 ± 9mg/dL) on entry to the study. Animal handling and anaesthesia protocols were optimized for MRI and are described elsewhere [8]. These procedures were developed in accordance with the recommendations of the Weather all report for performing non-human primate research [13]. Specifically, this study only used non-invasive, i.e. MRI, and minimally invasive, i.e. blood draws, data collection procedures, all of which were performed under general anaesthesia to ameliorate potential pain and suffering. The animal handling protocol and all procedures were reviewed and approved by the Institutional Animal Care and Use Committee of Texas Biomedical Research Institute.

### Peripheral Measurements

Blood was drawn from the right saphenous vein at 5 minute intervals for glucose measurements in all animals. In addition, 5 ml blood draws were performed at 0, 10, 20, 22.5, 27.5, 35, 45 and 55 min to ascertain glucose plasma concentrations in all five animals. Whole blood glucose was measured with a glucometer (Accuchek AVIVA, Roche Diagnostics).

### Magnetic resonance Imaging

Animals were fasted for 16 hours, with full access to water, before being transported from the SNPRC to the animal preparation area at the Research Imaging Institute (RII) at the University of Texas Health Science Center at San Antonio (UTHSCSA). All imaging experiments were performed using a Siemens TIM Trio 3T MRI scanner and an eight-channel phase-array body coil, as described elsewhere [14–15]. Each animal was sedated with an intramuscular injection of s-ketamine 10mg/kg (KetaVed, Phoenix Scientific, St. Joseph, Missouri), intubated with an MR-compatible tracheal tube and 18 gauge catheters were inserted into the left and right saphenous veins. Animals were then moved to the MRI room where anaesthesia was induced and maintained by mechanical ventilation, at the rate of 10 respiration/minute, with 2% Isoflurane. Animal’s heart rate, end-tidal CO_2_ concentrations and core body temperature were monitored using MRI compatible equipment for 15 min prior to imaging and throughout the imaging experiment. Each 60 minutes long MRI session consisted of 20 minutes of baseline imaging followed by a bolus injection of glucose (dextrose, 50%) calculated at 500 mg/kg of body weight into the left saphenous vein. The details of the body imaging experiment are discussed elsewhere [14–15]. In short, motion free images were obtained using respiratory gating signal from the anaesthesia machine. The entire upper body including the abdominal region was scanned using 3D, two-point Dixon fat-water separation imaging sequence with the isotropic resolution of (1mm3). The two-point Dixon technique collects two images, one where fat and water are in phase and one where they are out-of-phase [16]. Three hundred and twenty (320) slices were obtained in the superior-to-inferior field of view.

### Image processing

**Figure 1:**
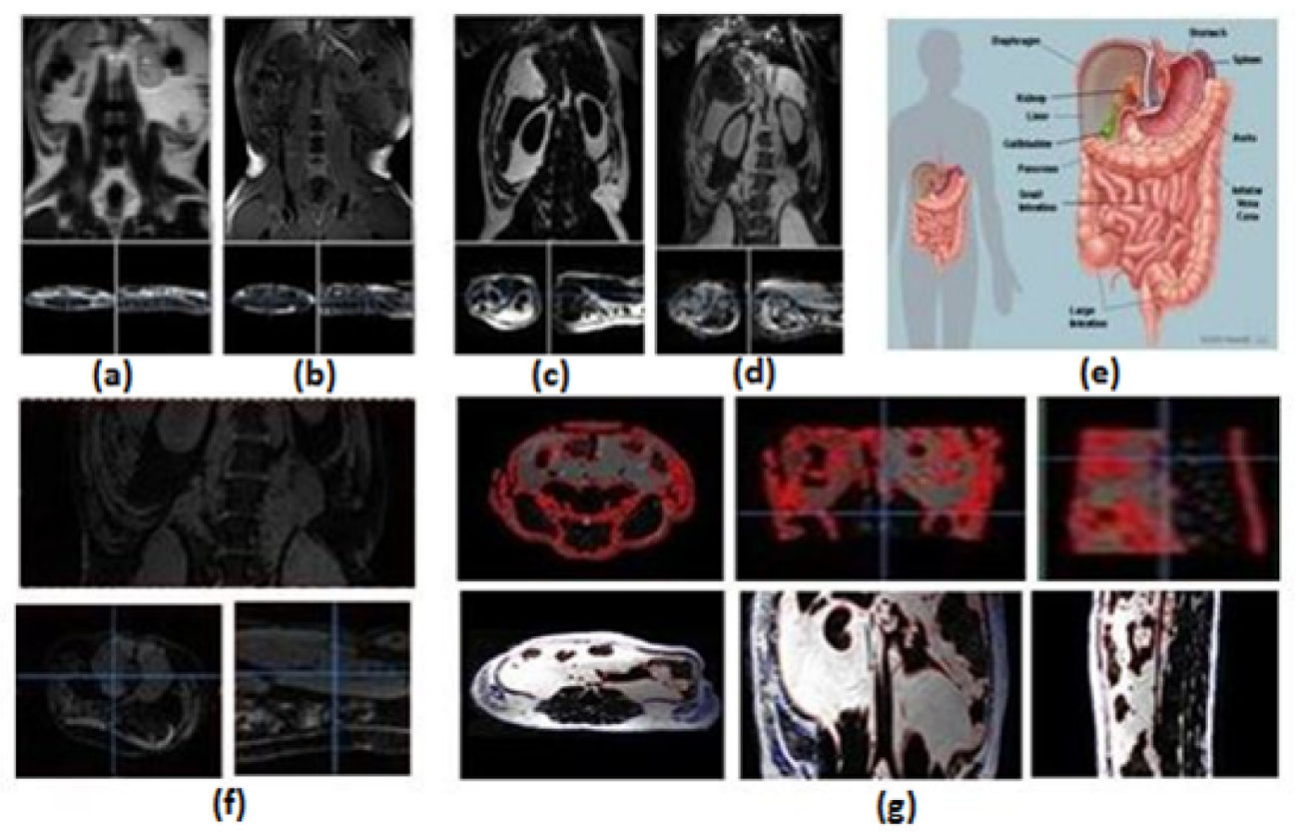
Stepwise Image processing. (a) In-phase raw image. (b) Out-of-phase raw image. (c)Fat image obtained by subtracting the raw images. (d)Muscle image obtained by adding the raw images. (e) Abdominal cavity. (f) Region of Interest-Lumbar vertebrae. (g) Processed Muscle and fat images.

Images were pre-processed using the Multi-Image Analysis GUI (MANGO) software (http://ric.uthscsa.edu/mango). The pre-processing steps included: correction for field inhomogeneity and quantification of tissue composition [16]. processed to obtain the spatial maps of the fat muscle distribution and then separate adipose tissues into the subcutaneous and the visceral fat constituents. Briefly, the raw image obtained via MRI scans comprised of fat and water components in-phase and out-of-phase (Figure 1a and 1b). Using the 2 point Dixon (2PD) method, the raw images was added and subtracted pixel-wise to generate water and fat images, respectively as shown in Figure 1c–1d. Figure 1g shows the separation of fat from the muscle content, which is highlighted in the red.

The abdominal cavity as illustrated in Figure 1e was defined as the space that corresponds to the lumbar spine segments. The Volume of Interest (VOI) was identified to be the lumbar vertebrae and is selected in the coronal view. The region outside VOI was set to zero as illustrated in Figure 1f. The VOI comprised of muscle adipose along with bones, tendons, cartilages which appears bright as well on the MRI scan. Each slice was manually processed to separate muscle adipose from bones, tendons, cartilages, etc. Figure 1g shows the separation of fat from the muscle content, which is highlighted in the red. The highlighted region represents the muscle region.

The fat image was then processed to separate subcutaneous and visceral fat partitions using peritoneal membrane. Prior to this, the fat image was then filtered with a size 3 x 3 x 3 mm median filter to improve separation between visceral and subcutaneous fat by eliminating connective bridges between subcutaneous and visceral fat components. We used a standard reference [17], to estimate the adipose tissue to be composed of 84.67% fat, 12.67% water, and 2.66% proteins and the density of the adipose tissue with the density of 0.9196 kg/l. The adipose tissue mass was calculated for each 3-mm slice. The total adipose tissue mass for each compartment were acquired from the cumulative masses for each slice.

### Statistical Analysis

A data-driven analysis approach was used to identify the effects of body fat composition on glucose perfusion, using the agent’s pharmacokinetic time-activity curve as a parametric statistical predictor of blood glucose levels. The agent’s time-activity curve is assessed by peripheral measurements of its concentration. We chose the blood glucose level as the predictor variable. Glucose level measurements require collection of only small amounts of blood (< 0.1 ml compared to 5 ml for measurements of regulatory hormones) and therefore can be performed frequently (every 5 min) for improved temporal sampling frequency.

The correlation analysis was performed using Pearson’s correlations. Polynomial regression was conducted where glucose responses was used as a dependent variable and fat deposition and body weight were used as independent variables. All results were considered significant analyses were performed using IBM SPSS Statistics 21 (IMB, Armonk, NY).

## Results

Average body fat composition in our NHP group was 68.4 ± 22.9%. All NHP subjects fit the criteria for abnormal body fat composition (> 65%). Tables 1 and 2 show the total adipose tissue mass and volume calculated, respectively. The adipose tissue compartments volume and mass varied from 0.318 L to 2.91 L and was the highest in the evaluation of subcutaneous fat for subject 3.

**Figure 2:**
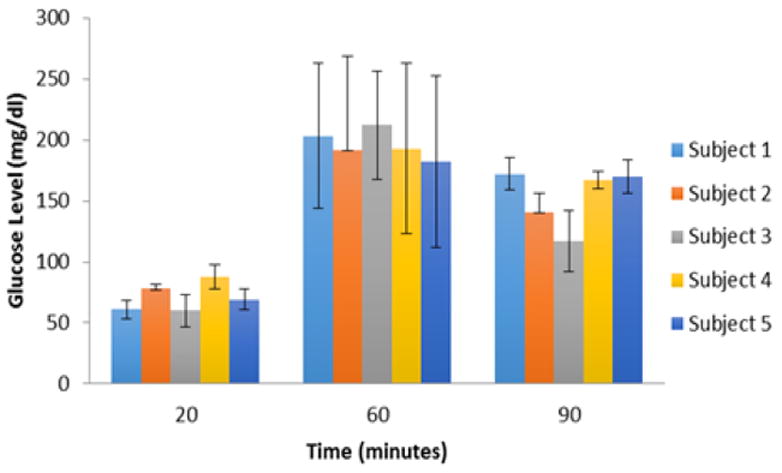
Glucose response in five female baboons.

**Figure 3:**
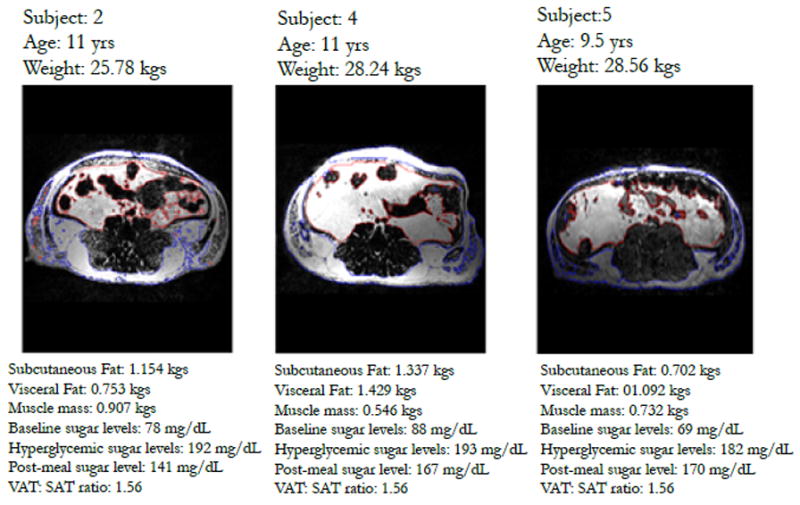
Three representative MRI abdominal fat images of baboon subjects. Note the increase in visceral fat, decrease in subcutaneous fat ratio across subjects and an increase in fasting glucose. (Blue region) subcutaneous fat and (Red region) visceral fat region.

The state of fasting glycemia was confirmed by measuring stable baseline blood glucose (51.3 ± 10.9 mg/dL). Infusion of glucose produced short (~20 min) lasting hyperglycemia (peak value = 203.9 ±38.9 mg/dL) (Figure 2). During the last twenty minutes, the blood glucose levels returned to normal post-meal values (134.4 ± 22.0 mg/dL). Pearson’s correlations showed a statistically-significant (p < 0.001) correlation between serum glucose concentration trends and muscle and visceral adipose tissue volume (Table 3). In addition, polynomial regression showed body weight, and visceral and muscle fat to be significant predictors of serum glucose response in these baboons. Figure 3 further illustrate three obese animals along with their metabolic profile associated with the partitioning in abdominal fat. Upon comparing the animals, animal 5 exhibits a higher ratio of visceral fat to subcutaneous fat and the reverse is observed in animal 3, along with the pronounced differences in the metabolic profile.

## Discussion

We combined MRI, time-resolved sampling of glucose concentrations and a data-driven analysis technique to study the effect of adipose tissue composition on the transition from fasting glycemia to hyperglycemia in NHP. We performed this study as a part of developing a non-human primate model to study the physiology and genetics of human metabolic disorders. Old-world NHP, such as baboons, are likely to provide relevant scientific models because they naturally develop many metabolic diseases observed in humans and therefore offer clinically relevant responses to therapeutic interventions. We aimed to study the relationship among adipose tissue compartments, specifically abdominal adipose tissue and the regulatory response to a large change in systemic glucose concentrations. An excessive accumulation of abdominal adipose tissue may have remarkably high metabolic activity. MRI was chosen to quantify total adipose tissue composition because it offered several advantages over computerized tomography in estimating the adipose tissue mass. A key advantage is the absence of radiation exposure and the ability to accurately acquire the volume of compartments. In addition, MRI enables easy identification of fat because fat has a short T1 and long T2 proton relaxation times as compared to other tissues [18–20] and adipose tissue is clearly visualized as bright areas that are contrasts with surrounding non-adipose tissues.

Our first aim was to measure the volume and mass of adipose tissue in NHPs by MRI and to evaluate the effect of these adipose tissue compartments on glucose responses via a hyperglycemic challenge. The abdominal adipose tissue has been reported to be metabolically active and no measurements are available on the quantification of the abdominal adipose tissue in NHPs in vivo. The measurement of the abdominal adipose tissue was extended to distinguish between the visceral and the subcutaneous adipose tissue volumes. The distinction between these two compartments is relevant because the anatomical vascular connections to the visceral space differ from the subcutaneous space. Both visceral and subcutaneous fat are metabolically different and therefore are easily distinguishable using MRI techniques.

The data obtained in this study indicates that not only can MRI be used for the determination of adipose tissue volume in the abdomen of NHPs, it can be used to separate visceral and subcutaneous adipose tissue volumes, as well as muscle volumes. The adipose tissue mass was estimated by taking the product of the total volume and the density of the adipose tissue for each baboon. Therefore, MRI appears to be a valuable method for the in vivo estimation of adipose tissue mass. The five baboons studied had a relative narrow weight range of 23–28 kg (Table 1), and the mass of adipose tissue in the visceral and subcutaneous compartments in the most obese baboon was relatively lower than that of the leanest subject. This raises the question of whether such relative small differences in the adipose masses could account for the major differences observed in metabolic complications associated with obesity. The ability to acquire masses in these adipose tissue compartments enabled the evaluation of the effect of the adipose tissue composition impact on a hyperglycemic challenge.

This study validates the use of MRI to study the adiposity in NHPs in vivo by enabling the evaluation of the different adipose tissue compartments, such as visceral and subcutaneous that is not typically accessible by the more conventional anthropometric measurements. The use of MRI in the determination of the distribution of adipose tissue in NHPs should improve our understanding of the impact of adiposity on the metabolic complications of obesity and diabetes.

## Figures and Tables

**Table 1:** Measured adipose tissue mass

	Adipose Tissue Mass (Kg)			
	Weight	Visceral	Subcutaneous	Muscle
Subject 1	23.25	1.376	1.100	0.642
Subject 2	25.78	0.753	1.154	0.907
Subject 3	27.68	1.269	2.680	1.110
Subject 4	28.24	1.429	1.337	0.546
Subject 5	28.56	1.092	0.702	0.732

Data are presented as total mass from slices.

**Table 2:** Measured adipose tissue volume

	Adipose Tissue Volume (l)		
	Visceral	Subcutaneous	Muscle
Subject 1	1.495	1.195	0.698
Subject 2	0.818	1.254	0.986
Subject 3	1.380	2.914	1.206
Subject 4	1.553	1.453	0.593
Subject 5	0.608	0.318	0.721

Data are presented as total volume from slices.

**Table 3:** Relationship between serum glucose response to hyperglycemic challenge and body weight, muscle fat and abdominal fat compartments

Trait1	Trait2	R2	P value

**Body weight**	Fasting glucose	0.99	<0.004
	Average glucose response (0–20min)	0.99	<0.003
	Average glucose response (20–60 min)	0.99	<0.0001
	Average glucose response (60–90min)	0.99	<0.0001
	Visceral adipose volume	0.97	<0.007
	Subcutaneous adipose volume	0.83	<0.068
	Muscle adipose volume	0.95	<0.012

**Visceral adipose**	Fasting glucose	0.28	0.65
	Average glucose response (0–20min)	−0.12	0.85
	Average glucose response (20–60min)	0.40	0.51
	Average glucose response (60–90min)	0.29	063

**Subcutaneous adipose**	Fasting glucose	0.53	0.35
	Average glucose response (0–20min)	−0.35	0.57
	Average glucose response (20–60min)	0.85	0.07
	Average glucose response (60–90min)	−0.85	0.07

**Total adipose volume**	Fasting glucose	0.27	0.66

**volume**	Average glucose response (0–20min)	0.92	0.03
	Average glucose response (20–60min)	−0.35	0.56
	Average glucose response (60–90min)	0.25	0.68
